# Treosulfan–fludarabine–thiotepa-based conditioning treatment before allogeneic hematopoietic stem cell transplantation for pediatric patients with hematological malignancies

**DOI:** 10.1038/s41409-020-0869-6

**Published:** 2020-03-20

**Authors:** Krzysztof Kalwak, Monika Mielcarek, Katharine Patrick, Jan Styczynski, Peter Bader, Selim Corbacioglu, Birgit Burkhardt, Karl Walter Sykora, Katarzyna Drabko, Jolanta Gozdzik, Franca Fagioli, Johann Greil, Bernd Gruhn, Rita Beier, Franco Locatelli, Ingo Müller, Paul Gerhardt Schlegel, Petr Sedlacek, Klaus Daniel Stachel, Claudia Hemmelmann, Ann-Kristin Möller, Joachim Baumgart, Ajay Vora

**Affiliations:** 1 Department of Pediatric Hematology, Oncology and Bone Marrow Transplantation, Wroclaw Medical University, Wroclaw, Poland; 2 Sheffield Children’s Hospital, Sheffield, UK; 3 Department of Pediatric Hematology and Oncology, Collegium Medicum UMK Torun, Bydgoszcz, Poland; 4 Department for Children and Adolescents, Division for Stem Cell Transplantation, Immunology and Intensive Care Medicine, Goethe University, Frankfurt, Germany; 5 University Hospital of Regensburg, Regensburg, Germany; 6 Department of Pediatric Hematology, Oncology and BMT, University Hospital Muenster, Muenster, Germany; 7 Department of Pediatrics, Hannover Medical School, Hannover, Germany; 8 Department of Pediatric Hematology, Oncology and Transplantology, Medical University of Lublin, Lublin, Poland; 9 Medical College, University Children’s Hospital in Cracow Jagiellonian University, Cracow, Poland; 10 Children’s Hospital Regina Margherita, University of Turin, Turin, Italy; 11 University Children’s Hospital Heidelberg, Heidelberg, Germany; 12 Department of Pediatrics, Jena University Hospital, Jena, Germany; 13 Depertment of Pediatrics III, University Hospital of Essen, Essen, Germany; 14 IRCCS Bambino Gesú Children’s Hospital, Sapienza University of Rome, Rome, Italy; 15 University Medical Center Hamburg-Eppendorf, Hamburg, Germany; 16 University Children’s Hospital of Wuerzburg, Wuerzburg, Germany; 17 Department of Pediatric Hematology and Oncology, University Hospital Motol, Prague, Czech Republic; 18 Children’s Hospital, University of Erlangen, Erlangen, Germany; 19 medac GmbH, Wedel, Germany; 20 Great Ormond Street Hospital, London, UK

**Keywords:** Leukaemia, Haematopoietic stem cells

## Abstract

Treosulfan-based conditioning prior to allogeneic transplantation has been shown to have myeloablative, immunosuppressive, and antineoplastic effects associated with reduced non-relapse mortality (NRM) in adults. Therefore, we prospectively evaluated the safety and efficacy of treosulfan-based conditioning in children with hematological malignancies in this phase II trial. Overall, 65 children with acute lymphoblastic leukemia (35.4%), acute myeloid leukemia (44.6%), myelodysplastic syndrome (15.4%), or juvenile myelomonocytic leukemia (4.6%) received treosulfan intravenously at a dose of 10 mg/m^2^/day (7.7%), 12 g/m^2^/day (35.4%), or 14 g/m^2^/day (56.9%) according to their individual body surface area in combination with fludarabine and thiotepa. The incidence of complete donor chimerism at day +28 was 98.4% with no primary and only one secondary graft failure. At 36 months, NRM was only 3.1%, while relapse incidence was 21.7%, and overall survival was 83.0%. The cumulative incidence of acute graft-vs.-host disease was 45.3% for grades I–IV and 26.6% for grades II–IV. At 36 months, 25.8% overall and 19.4% moderate/severe chronic graft-vs.-host disease were reported. These data confirm the safe and effective use of treosulfan-based conditioning in pediatric patients with hematological malignancies. Therefore, treosulfan/fludarabine/thiotepa can be recommended for myeloablative conditioning in children with hematological malignancies.

## Introduction

Children with hematological malignancies routinely undergo either busulfan- or fractionated TBI-based myeloablative conditioning regimens prior to allogenic hematopoietic stem cell transplantation (alloHSCT). However, both regimens are associated with a considerable risk of acute and late serious adverse events (AEs) [1–5]. Clinical studies, including prospective phase III trials in adults with AML and MDS, have shown that treosulfan-based conditioning has myeloablative, immunosuppressive, and antineoplastic effects associated with a low non-relapse mortality (NRM) [6–11]. Furthermore, several reports have been published that show an indication for treosulfan-based conditioning in children undergoing alloHSCT for nonmalignant and malignant disorders [12–21]. In addition, pharmacokinetic (PK) investigations on treosulfan in children have been conducted in order to derive dose recommendations for all pediatric age groups [22–25]. We therefore, prospectively evaluated treosulfan/fludarabine conditioning in pediatric patients with hematological malignancies during this extended clinical phase II trial. Herein, we strictly applied an individual body surface area (BSA)-adapted treosulfan dosing [26] with the option of administrating an intensified regimen by additional thiotepa infusion.

## Patients and methods

### Patient eligibility

Between November 2014 and July 2015, 70 children (aged 28 days to 17 years) with acute lymphoblastic leukemia (ALL), acute myeloid leukemia (AML), myelodysplastic syndrome (MDS), or juvenile myelomonocytic leukemia (JMML) were enrolled in this prospective non-randomized phase II trial at 18 transplantation sites in five European countries. Written informed consent on all aspects of the study was obtained from all children and/or their legal guardians before enrollment. The study was approved by the responsible independent ethics committees and competent authorities and was performed in accordance with the Declaration of Helsinki, as well as Good Clinical Practice guideline. Safety and outcome parameters of all surviving patients were analyzed after a 36 months follow-up period focusing on the 65 patients who were additionally treated with thiotepa. The 5 out of 70 patients, who received treosulfan and fludarabine only, were excluded from the statistical analysis reported here. However, the key findings for all 70 trial patients (including the five patients conditioned only with treosulfan/fludarabine) are provided in Fig. S1 in the online supplementary.

### Donors and grafts

Either human leucocyte antigens (HLA)-identical siblings (MSD), matched family donors, or matched unrelated donors were eligible donors (Table 1). An HLA match was defined as at least a 9/10 allele-matched after high-resolution four-digit typing in HLA-A*, B*, C* and DRB1* and DQB1*.

**Table 1:** Summary of demographic data and transplant characteristics by disease.

	Disease				
	ALL (*N* = 23)	AML (*N* = 29)	MDS (*N* = 10)	JMML (*N* = 3)	Overall (*N* = 65)
Sex [*n* (%)]					
Male	15 (65.2%)	19 (65.5%)	5 (50.0%)	3 (100.0%)	42 (64.6%)
Female	8 (34.8%)	10 (34.5%)	5 (50.0%)	0 (0.0%)	23 (35.4%)
Age [years]					
Mean (SD)	10.5 (5.6)	8.2 (5.6)	11.6 (5.7)	2.0 (2.0)	9.3 (5.8)
Median	12.0	8.0	14.0	2.0	11.0
Min., Max.	1, 17	0, 17	1, 17	0, 4	0, 17
ICH age group [*n* (%)]					
28 days to 23 months	2 (8.7%)	4 (13.8%)	1 (10.0%)	1 (33.3%)	8 (12.3%)
2–11 years	7 (30.4%)	14 (48.3%)	2 (20.0%)	2 (66.7%)	25 (38.5%)
12–17 years	14 (60.9%)	11 (37.9%)	7 (70.0%)	0 (0.0%)	32 (49.2%)
Number of HSCT [*n* (%)]					
1st	22 (95.7%)	28 (96.6)	8 (80)	2 (66.7%)	60 (92.3%)
2nd	1 (4.3%)	1 (3.4)	2 (20)	1 (33.3%)	5 (7.7%)
Number of complete remission [*n* (%)]^a^					
1. CR	16 (69.6%)	25 (86.2%)	na	na	41 (63.1%)
2. CR	7 (30.4%)	3 (10.3%)	na	na	10 (15.4%)
3. CR (or higher)	0 (0.0)	1 (3.4%)	na	na	1 (1.5%)
Secondary origin of malignancy [*n* (%)]					
Yes	1 (4.3)	0 (0.0)	4 (40)	0 (0.0)	5 (7.7)
Treosulfan dose [*n* (%)]					
10 g/m^2^/day −6, −5, −4	1 (4.3%)	3 (10.3%)	0 (0.0%)	1 (33.3%)	5 (7.7%)
12 g/m^2^/day −6, −5, −4	5 (21.7%)	13 (44.8%)	3 (30.0%)	2 (66.7%)	23 (35.4%)
14 g/m^2^/day −6, −5, −4	17 (73.9%)	13 (44.8%)	7 (70.0%)	0 (0.0%)	37 (56.9%)
Donor type [*n* (%)]					
Matched sibling	6 (26.1%)	4 (13.8%)	1 (10.0%)	0 (0.0%)	11 (16.9%)
Matched family	0 (0.0%)	1 (3.4%)	0 (0.0%)	0 (0.0%)	1 (1.5%)
Matched unrelated	17 (73.9%)	24 (82.8%)	9 (90.0%)	3 (100.0%)	53 (81.5%)
Source [*n* (%)]					
Bone marrow	14 (60.9%)	13 (44.8%)	3 (30.0%)	3 (100.0%)	33 (50.8%)
Peripheral blood	9 (39.1%)	16 (55.2%)	7 (70.0%)	0 (0.0%)	32 (49.2%)

*ALL* acute lymphoblastic leukaemia, *AML* acute myeloid leukaemia, *MDS* myelodysplastic syndrome, *JMML* juvenile myelomonocytic leukaemias, *ICH* International Council of Harmonization, *Max.* maximum, *Min.* minimum, *N* number of subjects, *n* number of subjects in category, *SD* standard deviation, *na* not applicable.

^a^For ALL and AML subjects only.

### Conditioning regimen and supportive care

All patients received an individualized BSA-adapted intravenous (IV) treosulfan dose on days −6 to −4, i.e., BSA of ≤0.5 m^2^ received 10 g/m^2^/day; of >0.5–1.0 m^2^ received 12 g/m^2^/day; and of >1.0 m^2^ received 14 g/m^2^/day. Subsequently, fludarabine 30 mg/m^2^/day IV was given from day −7 to day −3 (total dose: 150 mg/m^2^). At the investigator’s discretion, thiotepa 2 × 5 mg/kg/day IV was additionally given on day −2 to 65 patients (total dose: 10 mg/kg). Allogeneic hematopoietic stem cells obtained either from peripheral blood or from bone marrow were given at day 0. Supportive care, including GvHD prophylaxis and treatment, was performed according to center-specific guidelines.

### Endpoints and definitions

The objective of this phase II trial was to describe the safety and efficacy of treosulfan administered as part of a standardized fludarabine-containing conditioning treatment and to contribute to a PK model. Clinical endpoints included engraftment and complete donor-type chimerism (defined as ≥95% donor cells), NRM, disease relapse/progression (RI), relapse/progression-free survival (RFS/PFS), acute/chronic GvHD, GvHD-free and relapse/progression-free survival (GRFS) [27], cGvHD-free and relapse/progression-free survival (CRFS) [27], and overall survival (OS).

NRM is defined as the probability of dying without previous occurrence of a relapse/progression of the underlying disease. Relapse/progression and graft failures are considered competing events. For estimation of GRFS, events were defined as acute GvHD of at least grade III, moderate or severe chronic GvHD, and relapse/progression or death (whichever occurred first). For CGRFS, events were defined as moderate or severe chronic GvHD, relapse/progression or death (whichever occurred first). All AEs except hepatic sinusoidal obstruction syndrome (HSOS) and hepatic toxicities were based on the Common Terminology Criteria for Adverse Events (CTCAE, version 4.03) and evaluated until 100 days after alloHSCT. HSOS was evaluated according to Jones et al. [28] and hepatic toxicities according to Bearman [29]. Furthermore, treosulfan concentrations in plasma from each patient were analyzed to calculate PK parameters in a subset of patients. Blood samples were taken immediately after treosulfan infusion and thereafter within 15–30 min, 1–2 h, 3–6 h, and 7–8 h. Further details of bioanalytical methods and the model-based parameter calculation have been previously described [26, 30].

### Statistical considerations

Descriptive analyses were performed using frequency, mean, standard deviation, median, and range, as appropriate. All time to event endpoints were measured from the day of HSCT (except for cGvHD, which started 100 days after HSCT) to the time of event or competing event, if applicable. Patients alive without event (or competing event) were censored at the last follow-up or at day +100 after HSCT for aGvHD. Kaplan–Meier estimates were used for calculating OS, GRFS, and CRFS. Cumulative incidences were used for estimating NRM, RI, aGvHD, and conditional cumulative incidences for neutrophil/leukocyte/platelet engraftment. Competing events were as follows: relapse/progression and graft failure for NRM; deaths without relapse/progression and graft failure for RI; death, relapse/progression, and graft failure within 100 days after the end of HSCT for aGvHD; death, disease relapse/progression, or the use of rescue therapies for engraftment.

Any changes in single laboratory values were documented separately instead of being included in the AE analysis. Non-compartmental methodology was applied for the PK analysis of treosulfan. Based on the individual plasma concentration–time data, the following parameters were determined using the actual sampling times for treosulfan: *C*_max_, *t*_max_, AUC_last_, AUC_∞_, *t*_1/2_term, CL, and *V*_*d*_. Mean and standard deviations were calculated for all PK parameters, except for *t*_max_, for which the median and range were computed.

This trial was descriptive in nature, thus, *p* values (two sided) were explorative, based on a significance level of 0.1. The data were analyzed using Pearson chi-square test for chimerism, the log-rank test for OS, GRFS, and CRFS, and the Gray test for NRM, RI, a/cGvHD.

All analyses were predefined in the statistical analysis plan. SAS software (version 9.4; SAS, Cary, NC, USA) was used for statistical analyses.

## Results

### Demographics

Sixty-five pediatric Caucasian patients (aged 28 days to 17 years, median 11 years) with ALL [35.4%], AML [44.6%], MDS [15.4%], or JMML [4.6%] were conditioned with treosulfan, fludarabine, and thiotepa. Nearly all of the ALL and AML patients were in first or second complete remission and had received their first alloHSCT procedure. Patients with MDS were classified as having refractory anemia with excess blasts (50%) or refractory cytopenia (40%). Children received alloHSCT between November 2014 and July 2015. The median follow-up was 41.8 months (range for surviving patients: 24.2–57.5 months).

Depending on their individual BSA, patients received treosulfan IV at a dose of 10 g/m^2^/day (7.7%), 12 g/m^2^/day (35.4%), or 14 g/m^2^/day (56.9%). Treosulfan was combined with fludarabine and thiotepa in 65 of 70 patients at the investigators’ discretion (Table 1).

### Outcomes

#### Engraftment, graft failure, and chimerism

The number of patients achieving reconstitution of granulopoiesis was 64 (98.5%). One patient (1.4%) died before engraftment 15 days after alloHSCT. The maximum conditional cumulative incidence of engraftment reached was 100% (90% CI: 97.7, 100.0) 43 days after HSCT. No patient experienced primary graft failure, and one patient (1.4%) with ALL had decreased neutrophils and leukocytes counts, but presented 100% donor chimerism, for which the patient received a stem cell boost 113 days post transplant.

The incidence of complete donor-type chimerism at visit day +28 was 98.4% (90% CI: 92.8, 99.90), at visit day +100 the incidence was 92.2% (90% CI: 84.3, 96.9), and at 12 months the incidence was 92.6% (90% CI: 83.8, 97.4) (Table 2). In exploratory subgroup analyses, a statistically significant influence on complete chimerism of underlying disease (JMML with 0% at day +100), as well as 2nd alloHSCT procedure was recorded (day +100: *p* < 0.001 influence of underlying disease, *p* = 0.0566 2nd HSCT). In contrast, the treosulfan dose, donor type, or patients age group did not significantly influence the incidence of complete donor-type chimerism.

**Table 2:** Incidence of complete donor-type chimerism.

	Treosulfan
Subjects at risk at day +28 visit^a^	*N* = 64
Subjects with complete chimerism at day +28 visit [*n* (%)]	63 (98.4)
90% CI	(92.8, 99.9)
Subjects without information at day +28 visit [*n* (%)]	1 (1.6)
Subjects at risk at day +100 visit^a^	*N* = 64
Subjects with complete chimerism at day +100 visit [*n* (%)]	59 (92.2)
90% CI	(84.3, 96.9)
Subjects without information at day +100 visit [*n* (%)]	5 (7.8)
Subjects at risk at month 12 visit^a^	*N* = 54
Subjects with complete chimerism at month 12 visit [*n* (%)]	50 (92.6)
90% CI	(83.8, 97.4)
Subjects without information at month 12 visit [*n* (%)]	2 (3.7)

*CI* confidence interval, *N* number of subjects, *n* number of subjects in category.

^a^Subjects are at risk if they have a chimerism examination at the day +28, day +100, or month 12 visit or if they have survived day +30, day +107, or day +379.

#### Non-relapse mortality and relapse/progression incidence

**Fig. 1:**
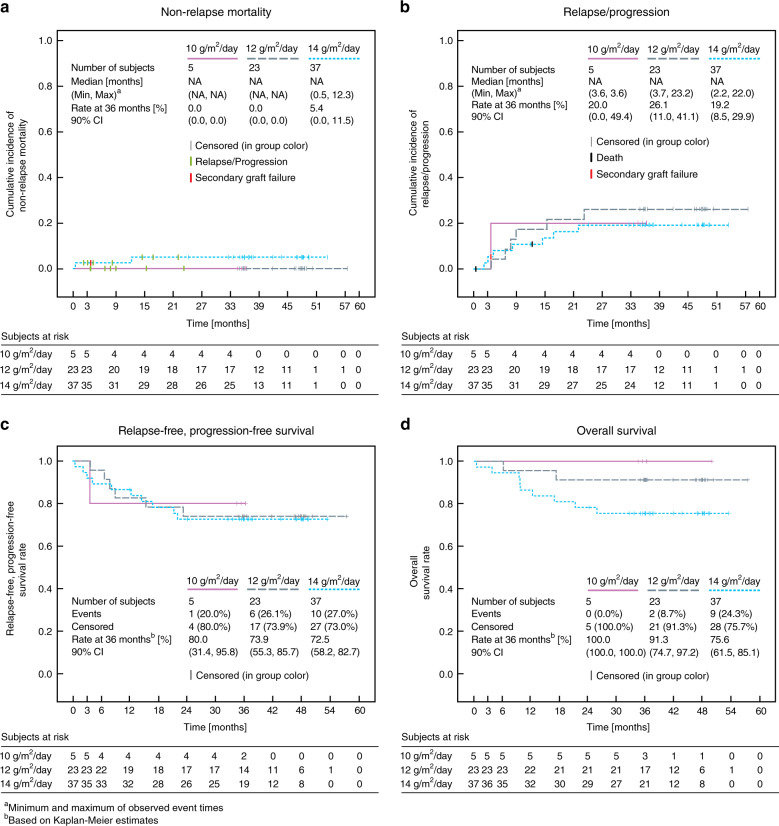
Treosulfan dose-dependent cumulative incidence of non-relapse mortality (**a**), relapse/progression (**b**), Kaplan–Meier estimate of relapse/progression-free survival (**c**) and of overall survival (**d**).

Overall, the cumulative incidence of NRM at 36 months was 3.1% (90% CI: 0.0, 6.6). One patient with AML died due to laryngeal hemorrhage 0.5 months after HSCT. A second patient with MDS died due to multi organ failure 12.3 months after HSCT. Due to the low number of events, no statistically significant difference in NRM was detected among the three different treosulfan dose groups (Fig. 1a) or within any other of the analyzed subgroups.

The cumulative incidence of relapse/progression (RI) at 36 months was 21.7% (90% CI: 13.2, 30.1). In the subgroup analyses, no statistically significant impact was recorded for treosulfan dose, donor type, or patient’s age group (Fig. 1b), but was found for underlying disease (*p* < 0.0001). Relapse incidences of ALL were 26.1%, AML 17.2%, MDS 0.0%, and JMML 100%. In the case of a 2nd HSCT procedure, the incidence of relapse/progression increased from 18.5 to 60.0% (*p* = 0.0140) considering that only five patients were treated in a second procedure.

#### Relapse/progression-free survival

**Fig. 2:**
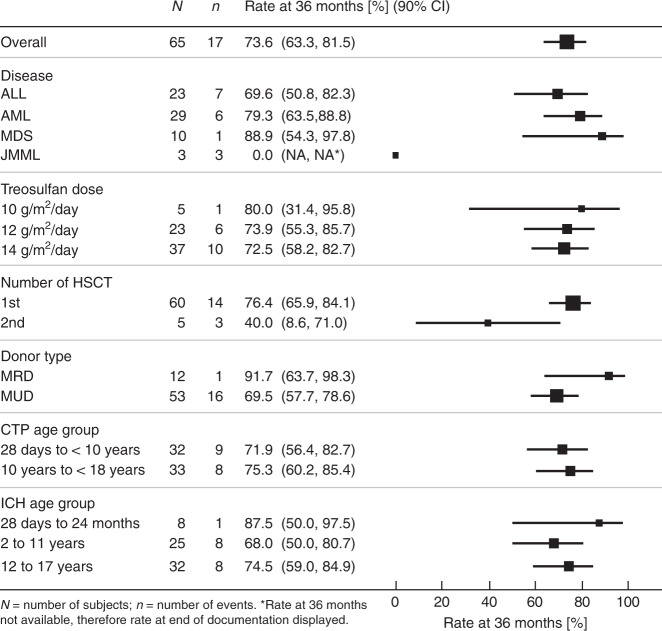
Forest plot for relapse/progression-free survival displaying 36 months rates by subgroups.

The 36-month Kaplan–Meier estimate of RFS/PFS was 73.6% (90% CI: 63.3, 81.5). In the exploratory subgroup analyses a statistically significant impact was recorded for 2nd HSCT (1st HSCT [76.4%]; 2nd HSCT [40%]; *p* = 0.0234) and underlying disease (ALL [69.6%], AML [79.3%], MDS [88.9%], JMML [0%]; *p* = 0.0001). However, in total only three JMML patients were included in the trial. There was no difference in RFS/PFS among donor types, age groups, and the three different treosulfan dose groups (Figs. 1c and 2).

#### Graft-vs.-host disease

At 100 days, the cumulative incidence of acute GvHD was: 45.3% (90% CI: 35.1, 55.5) grades I–IV, 26.6% (90% CI: 17.5, 35.6) grades II–IV, and 7.8% (90% CI: 2.3, 13.3) grades III–IV (Table 3). There was no difference among the three different treosulfan dose groups (Fig. S2 in online supplementary).

**Table 3:** Summary of cumulative incidence of acute and chronic GvHD.

aGvHD	Treosulfan (*N* = 65)
Grades I–IV	
Subjects with event [*n* (%)]	29 (44.6%)
Cumulative incidence of acute GvHD at 100 days (%)	45.3
90% CI	(35.1, 55.5)
Grades II–IV	
Subjects with event [*n* (%)]	17 (26.2%)
Cumulative incidence of acute GvHD at 100 days (%)	26.6
90% CI	(17.5, 35.6)
Grades III–IV	
Subjects with event [*n* (%)]	5 (7.7%)
Cumulative incidence of acute GvHD at 100 days (%)	7.8
90% CI	(2.3, 13.3)
Overall	
Subjects with event [*n* (%)]	16 (25.8%)
Cumulative incidence of cGvHD at 36 months (%)	25.8
90% CI	(16.7, 34.9)
Moderate/severe	
Subjects with event [*n* (%)]	12 (19.4%)
Cumulative incidence of cGvHD at 36 months (%)	19.4
90% CI	(11.1, 27.7)

*CI* confidence interval, *aGvHD* acute graft-vs.-host disease, *cGvHD* chronic graft-vs.-host disease, *N* number of subjects, *n* number of subjects in category.

The cumulative incidence of chronic GvHD at 36 months was 25.8% (90% CI: 16.7, 34.9) and that of moderate/severe chronic GvHD was 19.4% (90% CI: 11.1, 27.7) (Table 3 and Fig. S3 in the online supplementary). The ten MDS patients experienced a higher overall and moderate/severe chronic GvHD incidences (60.0% [95% CI: 34.5, 85.5] and 52% [90% CI: 25.0, 79.9], respectively).

#### GvHD-free and relapse/progression-free survival (GRFS) and cGvHD-free and relapse/progression-free survival (CRFS)

**Fig. 3:**
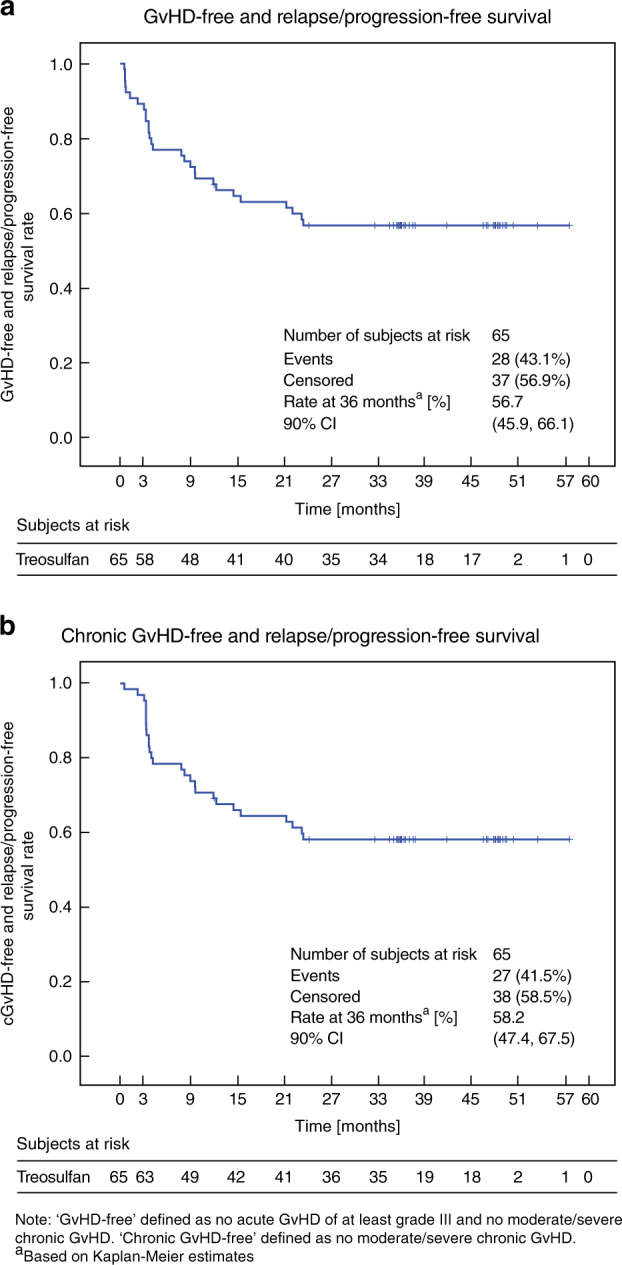
Kaplan–Meier estimates of (**a**) GvHD-free and relapse/progression-free survival and (**b**) chronic GvHD-free and relapse/progression-free survival.

The 36-month Kaplan–Meier estimate of GRFS was 56.7% (90% CI: 45.9, 66.1) (Fig. 3a). In the disease subgroups, GRFS was 56.5% at 36 months for ALL, 69.0% for AML, and 36.0% for MDS (JMML was not applicable). The five patients who underwent a second HSCT experienced a significantly lower incidence of GRFS with only 20.0% (90% CI: 1.8, 52.2; *p* = 0.0105) compared with patients after their first HSCT (59.7%; 90% CI: 48.5, 69.3).

The Kaplan–Meier estimate of CRFS at 36 months was 58.2% (90% CI: 47.4, 67.5) (Fig. 3b). In the disease subgroups, CRFS was 60.9% for ALL, 69.0% for AML, 36% for MDS, and 0.0% for JMML patients (*p* = 0.0017). Again, CRFS was worse after the second HSCT procedure (20.0 vs. 61.4%; *p* = 0.0060), while treosulfan dose, donor type, and age group did not influence this combined endpoint.

#### Overall survival (OS)

At the time of the final analysis, 11 patients (16.9%) had died. The causes of these deaths were relapse/progression related in eight (12.3%) patients, and transplantation related in three patients (4.6%).

**Fig. 4:**
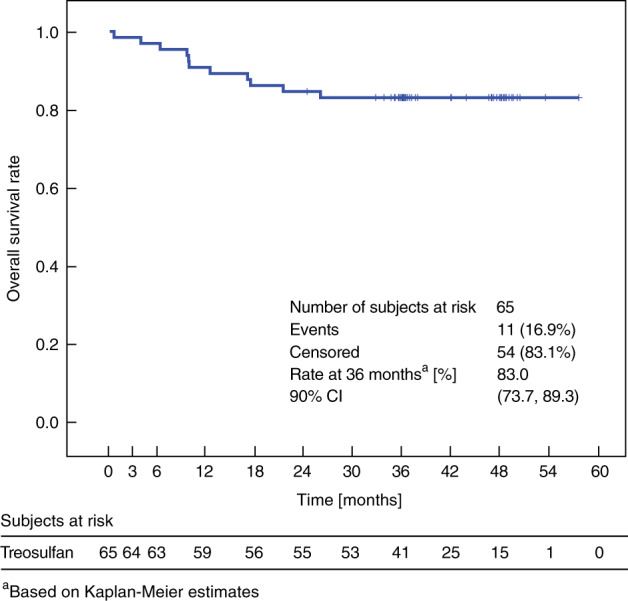
Kaplan–Meier estimate of overall survival.

The median time from transplantation to death was 9.72 months (Q1, Q3: 6.18, 17.25) with a range from 0.5 to 25.8 months. The 36-month Kaplan–Meier estimate of OS after HSCT was 83.0% (90% CI: 73.7, 89.3) (Fig. 4). OS estimates in ALL (78.3%), AML (86.2%), and MDS (90%) were comparable, while of the three JMML patients two survived. OS estimates in the ICH age groups were comparable to the treosulfan dose groups. Exploratory analysis indicated that there was a higher OS in eight patients aged 28 days to 23 months (100%, 90% CI: 100, 100) than in the 32 patients aged 12–17 years (74.9%, 90% CI: 59.5, 85.1). Accordingly (due to the individual BSA-related dose calculation), OS was 100% in the 10 g/m² treosulfan group and 75.6% (90% CI: 61.5, 85.1) in the 14 g/m² dose group (Fig. 1d). The five patients who received a 2nd HSCT had a lower OS probability at 36 months (60%, 90% CI: 19.1, 85.4) in comparison with the 60 patients who received a 1st alloHSCT procedure (85%, 90% CI: 75.4, 91.0).

#### Adverse events and pharmacokinetic results

Treatment-emergent AEs were reported in 63 of 65 patients (96.9%). The most common CTCAE terms with severity of at least grade III were mucositis—oral (43.1%), infections and infestations—other (30.8%), nausea and vomiting (both 16.9%), and diarrhea (15.4%) (Table 4). Skin and subcutaneous tissue disorders of at least CTCAE grade III were reported in 12.3% of the patients. One patient (1.4%) developed HSOS (grade II according to Jones et al. [28]) and recovered after 22 days.

**Table 4:** Frequency of patients with treatment emergent adverse events of at least CTCAE grade III in at least 10% of patients by System Organ Class and Preferred Term (MedDRA 20.0).

CTCAE System Organ Class CTCAE Term	Treosulfan (*N* = 65)
Subjects with any event	50 (76.9%)
Gastrointestinal disorders	
Any event	37 (56.9%)
Mucositis—oral	28 (43.1%)
Nausea	11 (16.9%)
Vomiting	11 (16.9%)
Diarrhea	10 (15.4%)
Dysphagia	2 (3.1%)
Abdominal pain	1 (1.5%)
Esophageal pain	1 (1.5%)
Typhlitis	1 (1.5%)
Upper gastrointestinal hemorrhage	1 (1.5%)
Infections and infestations	
Any event	28 (43.1%)
Infections and infestations—other, specify	20 (30.8%)
Catheter-related infection	6 (9.2%)
Sepsis	4 (6.2%)
Bladder infection	3 (4.6%)
Urinary tract infection	3 (4.6%)
Encephalitis infection	1 (1.5%)
Hepatitis viral	1 (1.5%)
Laryngitis	1 (1.5%)
Skin infection	1 (1.5%)
Soft tissue infection	1 (1.5%)
Upper respiratory infection	1 (1.5%)

Absolute and relative frequencies of subjects with event relative to the total number of subjects (*N*).

A total of 290 PK samples from 54 patients were analyzed. The mean AUC_last_ (±SD) was comparable among the three different dose groups (1686 ± 345 µg h/mL [10 g/m^2^], 1599 ± 33 µg h/mL [12 g/m^2^], and 1848 ± 283 µg h/mL [14 g/m^2^]) as evaluated in the PK subset of trial patients. In addition, the median *C*_max_ was comparable among the different dose or BSA groups (700 ± 218 µg/mL [10 g/m^2^], 634 ± 192 µg/mL [12 g/m^2^], and 650 ± 98 µg/mL [14 g/m^2^]) reflecting the individualized dose calculation based on BSA (Table 5). However, treosulfan plasma clearance and volume of distribution increased in the different dose groups. This observation is most likely based on the different ages, and BSA of the patients represented within the different dose groups.

**Table 5:** Pharmacokinetic results of treosulfan stratified by BSA-dependent dose group (non-compartmental analysis).

Mean ± SD, *t*_max_: median (range)	10 g/m²	12 g/m²	14 g/m²
*N*	5	23^a^	26
*C*_max_ [µg/mL]	700 ± 218	634 ± 192	650 ± 98
*t*_max_ [h]	2.28 (2.00–2.30)	2.00 (2.00–2.50)	2.02 (2.00–2.65)
AUC_last_ [µg h/mL]	1686 ± 345	1599 ± 33	1848 ± 283
AUC_∞_ [µg h/mL]	1700 ± 351	1627 ± 344	1900 ± 296
*t*_1/2term_ [h]	1.15 ± 0.12	1.38 ± 0.21	1.59 ± 0.18
CL [L/h]	2.33 ± 0.60	5.3 ± 1.35	10.94 ± 2.41
*V*_*d*_ [L]	3.86 ± 1.11	10.78 ± 3.69	25.24 ± 6.64

^a^*N* = 25 for AUC_∞_, t_1/2term_, CL, *V*_*d*_*.*

*SD* standard deviation, *t*_ma_ time to reach maximum plasma concentration, *N* number of subjects, *C*_max_ maximum plasma concentration, *h* hour, *AUC* area under the curve, *AUC*_las_ AUC from time 0 to the time of the last measurable plasma concentration, *AUC*_∞_ AUC from time 0 to infinite time, *t*_1/2term_ apparent terminal elimination half-life, *CL* total clearance; *V*_*d*_ volume of distribution.

## Discussion

After initial data on treosulfan-based conditioning in adult alloHSCT patients became available [9, 10], several published studies reported on the efficacy and safety of treosulfan given as part of various conditioning regimens in children [12, 13, 15, 16, 20, 31]. Here, we report the final results of the first prospective alloHSCT trial of a treosulfan/fludarabine/thiotepa combination regimen (FTT; 65 of 70 trial patients treated) administered to children and adolescents with hematological malignancies. In this study, an individual BSA-adapted dose calculation was consistently applied for treosulfan.

The 36-month Kaplan–Meier estimates of 3.1% for NRM, 83.0% for OS, and 73.6% for RFS/PFS compare favorably with the survival data reported for other conditioning regimens [2–5]. Overall, the 36-month relapse rate was low (21.7%), although three patients with JMML relapsed between 6 and 9 months post transplant. Disease recurrence is known to be particularly high in patients with JMML given an allograft, but the percentage of relapse seems to be higher than that reported using the busulfan/cyclophosphamide/melphalan regimen [32]. However, due to the very low number of JMML patients, no conclusions can be drawn at this point in time. On the other hand, the 36-month relapse incidence of 26.1% for the ALL subgroup (23 patients) is probably comparable to that of standard TBI-based regimens. Numerically, it might be slightly higher compared with the data published by Peters et al. [2]. The value of TBI-based and chemotherapy-based conditioning in childhood ALL is currently being investigated in an international controlled phase III trial (EudraCT No.: 2012–003032–22; ClinicalTrials.gov Identifier: NCT01949129). The relapse incidence of 17.2% at 36 months for the 29 AML patients and of 0% for the 10 MDS patients compares favorably with that reported for busulfan-based conditioning treatments [2, 33, 34]. Excellent results of the FTT regimen in children with AML (low relapse rate and reduced toxicity) warrant assessing this regimen in a prospective randomized pediatric alloHSCT trial in Europe, comparing busulfan- and treosulfan-based conditioning regimens for AML.

All patients achieved neutrophil engraftment and 98.4% of patients had a complete donor chimerism by day +28 post transplant. While neutrophil engraftment was comparable to that of previous reports [12, 20, 31], no prospective trial data on chimerism have been previously reported in children with malignant disorders who received conditioning with FTT. Hence, this study provides confirmation of the myeloablative potential of a FTT conditioning regimen, which results in an excellent rate of complete donor-type chimerism.

There were no primary graft failures. One ALL patient developed secondary poor graft function with decreasing blood cell counts associated with a viral illness despite 100% donor chimerism and received a stem cell boost.

The incidence of liver toxicity was relatively low with only one case (1.5%) of grade II HSOS, which resolved under appropriate therapy, compared with an up to 22% HSOS incidence reported in busulfan-containing regimens [3, 35]. Moreover, skin and subcutaneous tissue disorders of at least CTCAE grade III were within an acceptable range (12.3%) despite the intensification of the regimen with the addition of thiotepa. Just over half of the patients developed gastrointestinal (GI) AEs of at least CTCAE grade III, while oral mucositis was most prominent (43.1%). Compared with the data published by Boztug et al. [20], GI toxicity was relatively frequent, but not life-threatening, and was probably promoted by the addition of thiotepa in the vast majority of patients.

Unlike previous reports [22–25], we observed a rather limited variability in interindividual PKs of treosulfan. The BSA-banded dose calculation applied in this trial achieved equivalent treosulfan exposure (AUC, *C*_max_) in all dose groups. In the dose subgroups, the differences in outcome parameters, such as overall survival, were therefore, probably not due to a higher treosulfan exposure, but may be related to specific patient-, graft-, and/or underlying disease characteristics. The wide age range of patients included in this trial (from 28 days to 17 years of age) is reflected by the increase in treosulfan plasma clearance and volume of distribution within the different dose groups. Apparently, this observation is not related to treosulfan dose, but rather to the age-related differences in organ function maturation that are taken into account in the BSA-dependent dose calculation. Therefore, the strict application of the individual BSA-dependent treosulfan dose calculation should remain valid for the pediatric population [26].

There are several limitations to this prospective phase II study. Only a small number of patients were treated, especially in the MDS and JMML subgroups. Consequently, the poor outcome reported for JMML patients should be treated with caution. However, at least one JMML patient was considered at increased risk due to his second alloHSCT procedure.

The NRM, incidence of early toxicities, and donor chimerism, though, are likely to remain stable beyond this reported 36-month observation period. We can thus, safely conclude that FTT conditioning achieves high rates of engraftment with relatively low toxicity. Based on these and other clinical data, the European Commission has most recently approved treosulfan for conditioning in pediatric patients older than 1 month with malignant diseases [36].

We conclude that treosulfan-based conditioning with BSA-adapted dosing is safe and effective in pediatric patients with hematological malignancies. The cumulative incidences of OS and NRM compare favorably with those reported for other conditioning regimens. Treosulfan/fludarabine/thiotepa is, therefore, recommended as a suitable myeloablative preparative treatment for this pediatric patient population.

## Supplementary information

Supplementary Figure Legends

Figure S1

Figure S2

Figure S3

## References

[1] Niemeyer CM, Kratz CP (2008). Paediatric myelodysplastic syndromes and juvenile myelomonocytic leukaemia: molecular classification and treatment options. Br J Haematol.

[2] Peters C, Schrappe M, Stackelberg A, von Schrauder A, Bader P, Ebell W (2015). Stem-cell transplantation in children with acute lymphoblastic leukemia: a prospective international multicenter trial comparing sibling donors with matched unrelated donors-The ALL-SCT-BFM-2003 trial. J Clin Oncol.

[3] Beier R, Albert MH, Bader P, Borkhardt A, Creutzig U, Eyrich M (2013). Allo-SCT using BU, CY and melphalan for children with AML in second CR. Bone Marrow Transplant.

[4] Balduzzi A, Valsecchi MG, Silvestri D, Locatelli F, Manfredini L, Busca A (2002). Transplant-related toxicity and mortality: an AIEOP prospective study in 636 pediatric patients transplanted for acute leukemia. Bone Marrow Transplant.

[5] Bresters D, van Gils ICM, Kollen WJW, Ball LM, Oostdijk W, van der Bom JG (2010). High burden of late effects after haematopoietic stem cell transplantation in childhood: a single-centre study. Bone Marrow Transplant.

[6] Shimoni A, Labopin M, Savani B, Hamladji R-M, Beelen D, Mufti G (2018). Intravenous busulfan compared with treosulfan-based conditioning for allogeneic stem cell transplantation in acute myeloid leukemia: a study on behalf of the acute leukemia working party of european society for blood and marrow transplantation. Biol Blood Marrow Transplant.

[7] Nagler A, Labopin M, Beelen D, Ciceri F, Volin L, Shimoni A (2017). Long-term outcome after a treosulfan-based conditioning regimen for patients with acute myeloid leukemia: a report from the Acute Leukemia Working Party of the European Society for Blood and Marrow Transplantation. Cancer.

[8] Ruutu T, Volin L, Beelen DW, Trenschel R, Finke J, Schnitzler M (2011). Reduced-toxicity conditioning with treosulfan and fludarabine in allogeneic hematopoietic stem cell transplantation for myelodysplastic syndromes: final results of an international prospective phase II trial. Haematologica.

[9] Kröger N, Bornhäuser M, Stelljes M, Pichlmeier U, Trenschel R, Schmid C (2015). Allogeneic stem cell transplantation after conditioning with treosulfan, etoposide and cyclophosphamide for patients with ALL: a phase II-study on behalf of the German Cooperative Transplant Study Group and ALL Study Group (GMALL). Bone Marrow Transplant.

[10] Casper J, Knauf W, Kiefer T, Wolff D, Steiner B, Hammer U (2004). Treosulfan and fludarabine: a new toxicity-reduced conditioning regimen for allogeneic hematopoietic stem cell transplantation. Blood.

[11] Beelen DW, Trenschel R, Stelljes M, Groth C, Masszi T, Reményi P, et al. Treosulfan or busulfan plus fludarabine as conditioning treatment before allogeneic haemopoietic stem cell transplantation for older patients with acute myeloid leukaemia or myelodysplastic syndrome (MC-FludT.14/L): a randomised, non-inferiority, phase 3 trial. Lancet Haematol. 2019:e28–39. 10.1016/S2352-3026(19)30157-7. 31606445

[12] Slatter MA, Rao K, Abd Hamid IJ, Nademi Z, Chiesa R, Elfeky R (2018). Treosulfan and fludarabine conditioning for hematopoietic stem cell transplantation in children with primary immunodeficiency: UK experience. Biol Blood Marrow Transplant.

[13] Burroughs LM, Shimamura A, Talano J-A, Domm JA, Baker KK, Delaney C (2017). Allogeneic hematopoietic cell transplantation using treosulfan-based conditioning for treatment of marrow failure disorders. Biol Blood Marrow Transplant.

[14] Marzollo A, Calore E, Tumino M, Pillon M, Gazzola MV, Destro R (2017). Treosulfan-based conditioning regimen in sibling and alternative donor hematopoietic stem cell transplantation for children with sickle cell disease. Mediterr J Hematol Infect Dis.

[15] Morillo-Gutierrez B, Beier R, Rao K, Burroughs L, Schulz A, Ewins A-M (2016). Treosulfan-based conditioning for allogeneic HSCT in children with chronic granulomatous disease: a multicenter experience. Blood.

[16] Slatter MA, Boztug H, Pötschger U, Sykora K-W, Lankester A, Yaniv I (2015). Treosulfan-based conditioning regimens for allogeneic haematopoietic stem cell transplantation in children with non-malignant diseases. Bone Marrow Transplant.

[17] Strocchio L, Zecca M, Comoli P, Mina T, Giorgiani G, Giraldi E (2015). Treosulfan-based conditioning regimen for allogeneic haematopoietic stem cell transplantation in children with sickle cell disease. Br J Haematol.

[18] Dinur-Schejter Y, Krauss AC, Erlich O, Gorelik N, Yahel A, Porat I (2015). Bone marrow transplantation for non-malignant diseases using treosulfan-based conditioning. Pediatr Blood Cancer.

[19] Maschan M, Shelikhova L, Ilushina M, Kurnikova E, Boyakova E, Balashov D (2016). TCR-alpha/beta and CD19 depletion and treosulfan-based conditioning regimen in unrelated and haploidentical transplantation in children with acute myeloid leukemia. Bone Marrow Transplant.

[20] Boztug H, Sykora K-W, Slatter M, Zecca M, Veys P, Lankester A (2016). European Society for blood and marrow transplantation analysis of treosulfan conditioning before hematopoietic stem cell transplantation in children and adolescents with hematological malignancies. Pediatr Blood Cancer.

[21] Bernardo ME, Piras E, Vacca A, Giorgiani G, Zecca M, Bertaina A (2012). Allogeneic hematopoietic stem cell transplantation in thalassemia major: results of a reduced-toxicity conditioning regimen based on the use of treosulfan. Blood.

[22] van der Stoep MYEC, Bertaina A, Brink MHten, Bredius RG, Smiers FJ, Wanders DCM (2017). High interpatient variability of treosulfan exposure is associated with early toxicity in paediatric HSCT: a prospective multicentre study. Br J Haematol.

[23] Romański M, Wachowiak J, Główka FK (2018). Treosulfan pharmacokinetics and its variability in pediatric and adult patients undergoing conditioning prior to hematopoietic stem cell transplantation: current state of the art, in-depth analysis, and perspectives. Clin Pharmacokinet.

[24] Chiesa R, Standing JF, Winter R, Nademi Z, Chu J, Pinner D, et al. Proposed therapeutic range of treosulfan in reduced toxicity pediatric allogeneic hematopoietic stem cell transplant conditioning: results from a prospective trial. Clin Pharmacol Ther. 2019. 10.1002/cpt.1715. 10.1002/cpt.1715 PMC7484914 31701524

[25] van der Stoep MYEC, Zwaveling J, Bertaina A, Locatelli F, Guchelaar HJ, Lankester AC (2019). Population pharmacokinetics of treosulfan in paediatric patients undergoing hematopoietic stem cell transplantation. Br J Clin Pharm.

[26] Reijmers T, Hemmelmann C, Sykora K-W, Vora A, Kehne J, Möller A-K, et al. Population PK-modelling of treosulfan in paediatric allogeneic transplant patients. Twenty-seventh Annual Meeting of the Population Approach Group in Europe (page), Montreux; 2018.

[27] McCurdy SR, Kasamon YL, Kanakry CG, Bolaños-Meade J, Tsai H-L, Showel MM (2017). Comparable composite endpoints after HLA-matched and HLA-haploidentical transplantation with post-transplantation cyclophosphamide. Haematologica.

[28] Jones RJ, Lee KS, Beschorner WE, Vogel VG, Grochow LB, Braine HG (1987). Venoocclusive disease of the liver following bone marrow transplantation. Transplantation.

[29] Bearman SI, Appelbaum FR, Buckner CD, Petersen FB, Fisher LD, Clift RA (1988). Regimen-related toxicity in patients undergoing bone marrow transplantation. J Clin Oncol.

[30] Maiolica A, Meunier L, Bialleck S, Guhde I, Wood S, Struwe P. Bioanalytical determination of treosulfan and its active metabolites. EBF 7th Open Meeting, Barcilona; 2014.

[31] Nemecek ER, Hilger RA, Adams A, Shaw BE, Kiefer D, Le-Rademacher J (2018). Treosulfan, fludarabine, and low-dose total body irradiation for children and young adults with acute myeloid leukemia or myelodysplastic syndrome undergoing allogeneic hematopoietic cell transplantation: prospective phase II trial of the pediatric blood and marrow transplant consortium. Biol Blood Marrow Transplant.

[32] Locatelli F, Nöllke P, Zecca M, Korthof E, Lanino E, Peters C (2005). Hematopoietic stem cell transplantation (HSCT) in children with juvenile myelomonocytic leukemia (JMML): results of the EWOG-MDS/EBMT trial. Blood.

[33] Lucchini G, Labopin M, Beohou E, Dalissier A, Dalle JH, Cornish J (2017). Impact of conditioning regimen on outcomes for children with acute myeloid leukemia undergoing transplantation in first complete remission. An analysis on behalf of the pediatric disease working party of the European Group for Blood and Marrow Transplantation. Biol Blood Marrow Transplant.

[34] Strahm B, Nöllke P, Zecca M, Korthof ET, Bierings M, Furlan I (2011). Hematopoietic stem cell transplantation for advanced myelodysplastic syndrome in children: results of the EWOG-MDS 98 study. Leukemia.

[35] Bartelink IH, van Reij EML, Gerhardt CE, van Maarseveen EM, Wildt A, de, Versluys B (2014). Fludarabine and exposure-targeted busulfan compares favorably with busulfan/cyclophosphamide-based regimens in pediatric hematopoietic cell transplantation: maintaining efficacy with less toxicity. Biol Blood Marrow Transplant.

[36] European Medicines Agency. Union register of medicinal products: marketing authorisation. Decision number (2019)4858 of 20 Jun 2019. 2019. https://ec.europa.eu/health/documents/community-register/html/h1351.htm.

