# Supplementary material for: Treosulfan–fludarabine–thiotepa-based conditioning treatment before allogeneic hematopoietic stem cell transplantation for pediatric patients with hematological malignancies
Source: Bone Marrow Transplant. 2020 Mar 20;55(10):1996–2007. doi: 10.1038/s41409-020-0869-6 (PMC7515850; doi:10.1038/s41409-020-0869-6)
Supplement: Supplementary file 1 — Supplementary Figure Legends [file 41409_2020_869_MOESM1_ESM.docx]

**Supplementary Figure Legends**

**Figure S1 Cumulative incidence of Non-relapse mortality (A), Relapse/progression (B), Kaplan-Meier estimate of Relapse/progression-free survival (C) and of Overall survival (D) Treosulfan dose-dependent – all 70 patients**

**Figure S2 Cumulative incidence of aGvHD grade III – IV by Treosulfan dose**

**Figure S3 Cumulative incidence of moderate/severe GvHD by Treosulfan dose**
