# Supplementary material for: Treosulfan–fludarabine–thiotepa-based conditioning treatment before allogeneic hematopoietic stem cell transplantation for pediatric patients with hematological malignancies
Source: Bone Marrow Transplant. 2020 Mar 20;55(10):1996–2007. doi: 10.1038/s41409-020-0869-6 (PMC7515850; doi:10.1038/s41409-020-0869-6)
Supplement: Supplementary file 2 — Figure S1 [file 41409_2020_869_MOESM2_ESM.pdf]

FIGURE S1 **Cumulative incidence of Non-relapse mortality (A), Relapse/Progression (B), Kaplan-Meier estimate of Relapse/progression-free survival (C), and of overall survial (D) Treosulfan dose-dependent - of all 70 patients**

A. Non-relapse mortality

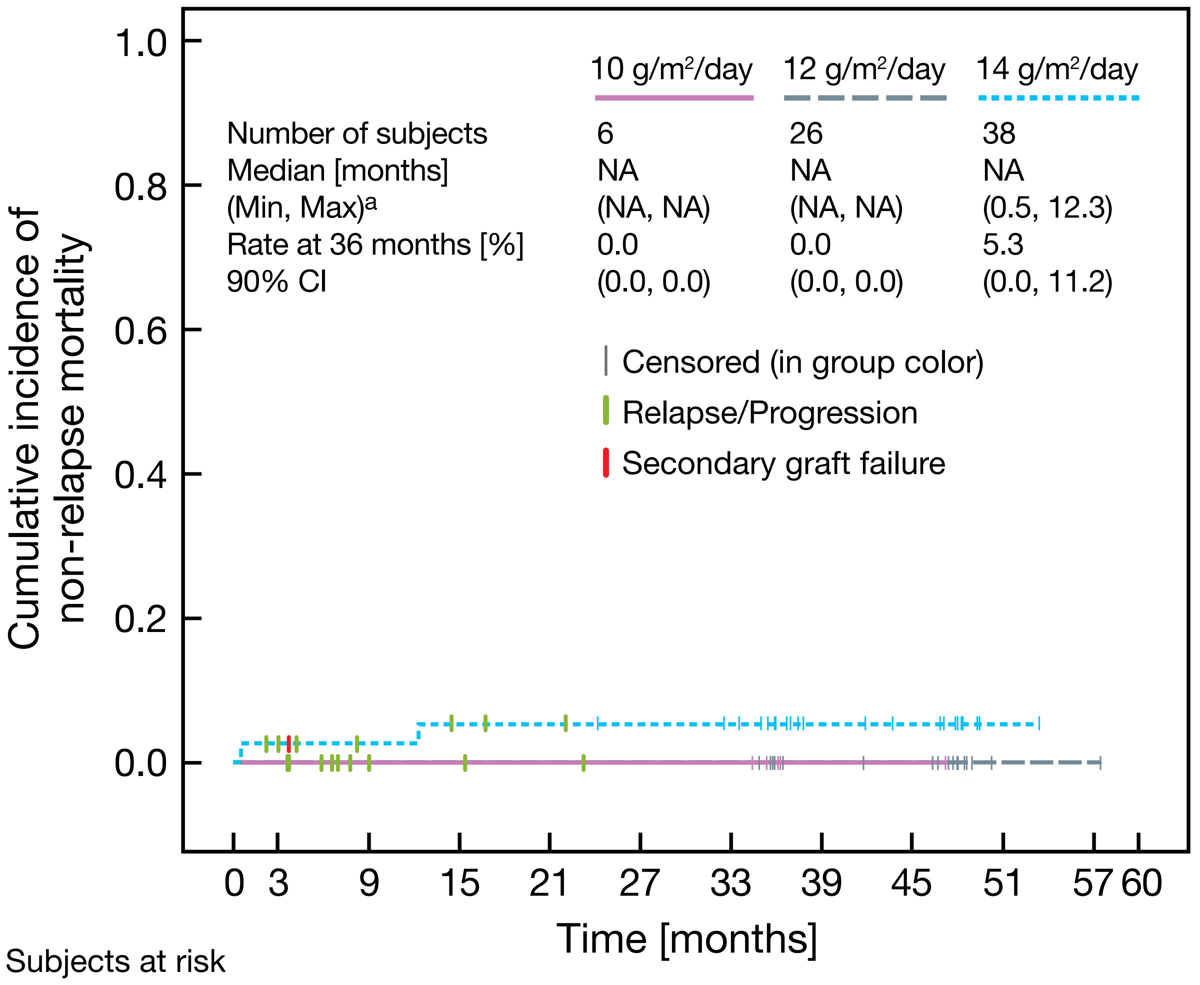

Subjects at risk

10 g/m²/day	6	6	5	5	5	5	5	1	1	0	0	0
12 g/m²/day	26	26	21	20	19	18	18	12	11	1	1	0
14 g/m²/day	38	36	32	30	29	27	26	13	11	1	0	0

B. Relapse/progression

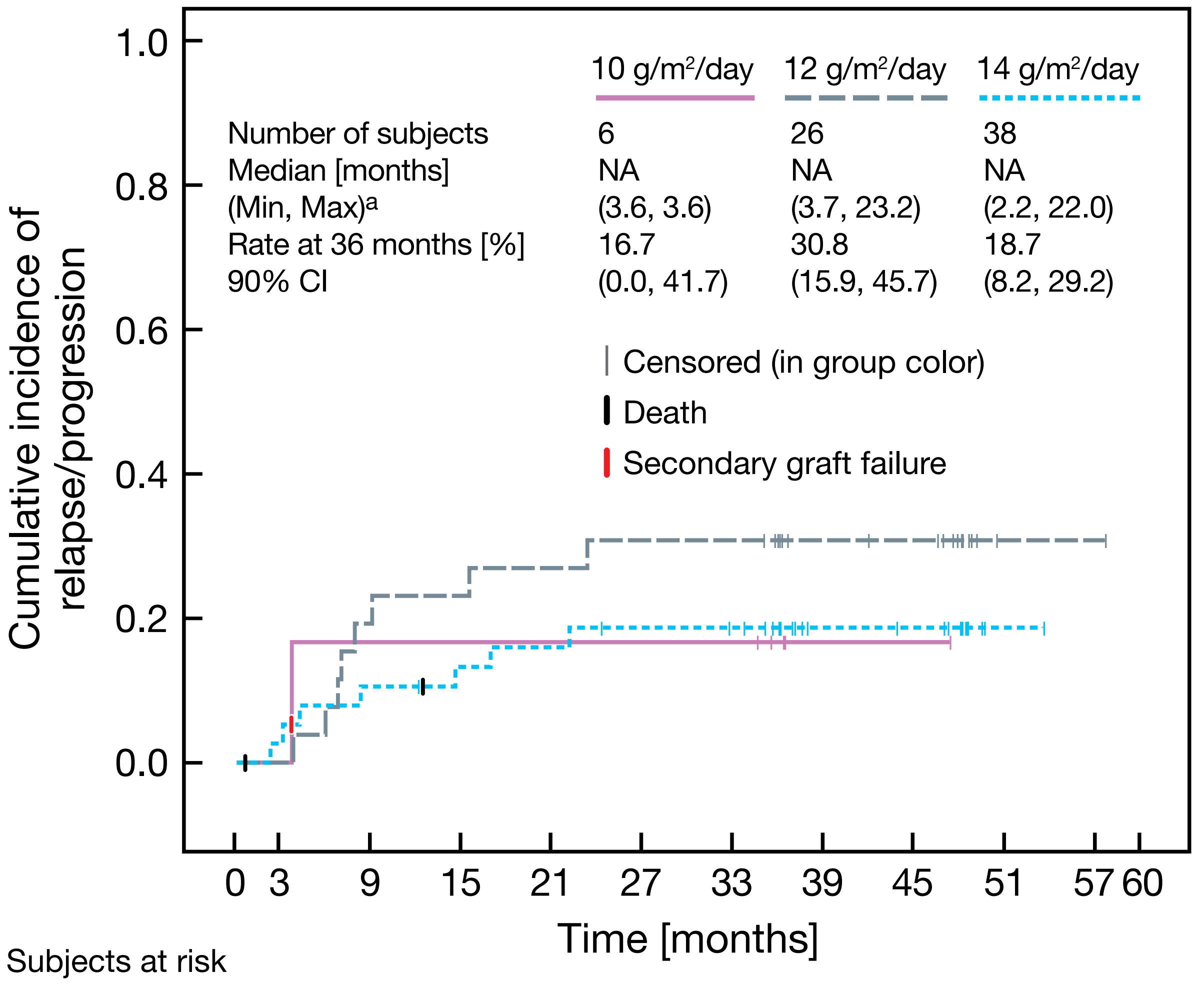

Subjects at risk

10 g/m²/day	6	6	5	5	5	5	5	1	1	0	0	0
12 g/m²/day	26	26	21	20	19	18	18	12	11	1	1	0
14 g/m²/day	38	36	32	29	28	26	25	12	11	1	0	0

C. Relapse-free, progression-free survival

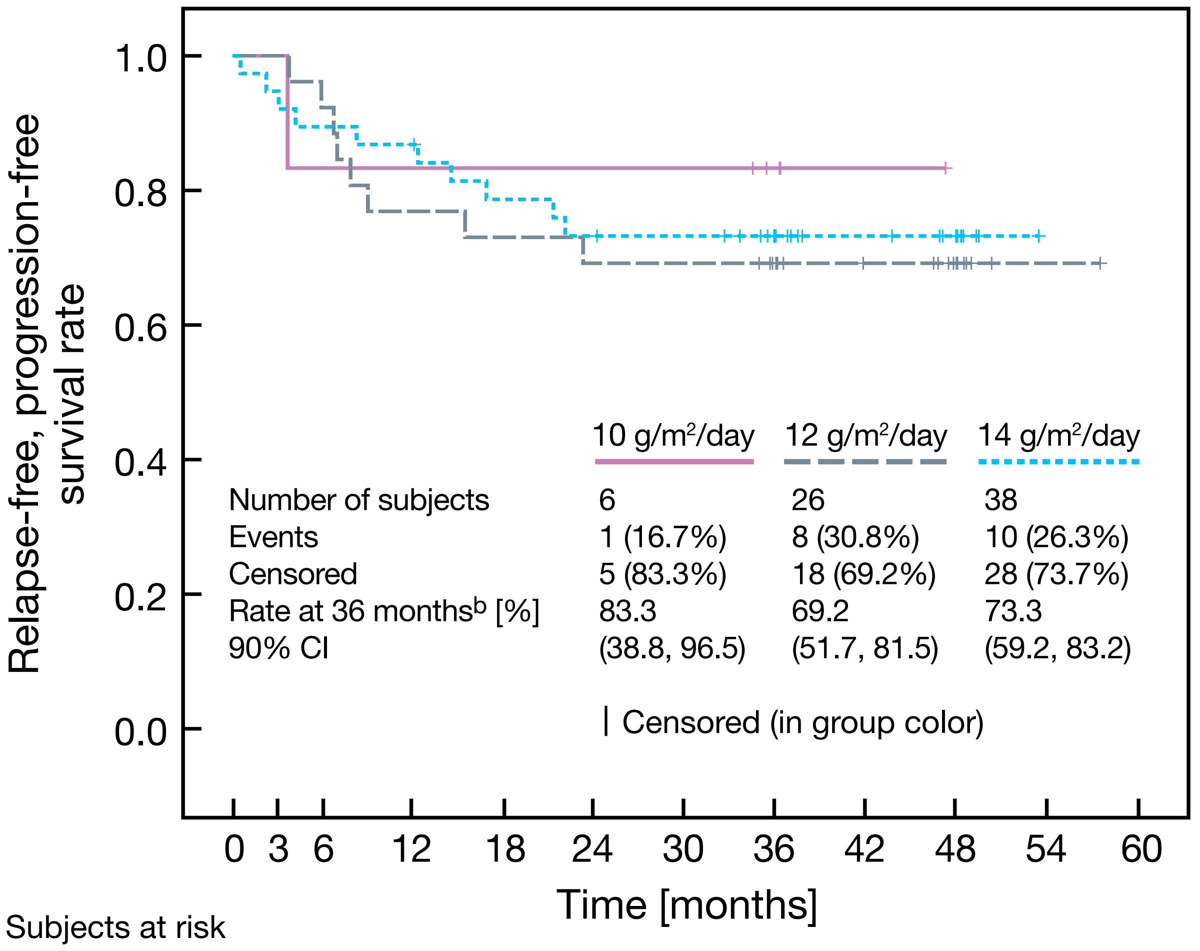

Subjects at risk

10 g/m²/day	6	6	5	5	5	5	5	3	0	0	0	0
12 g/m²/day	26	26	24	20	19	18	18	15	11	6	1	0
14 g/m²/day	38	36	34	33	29	27	26	19	12	8	0	0

D. Overall survival

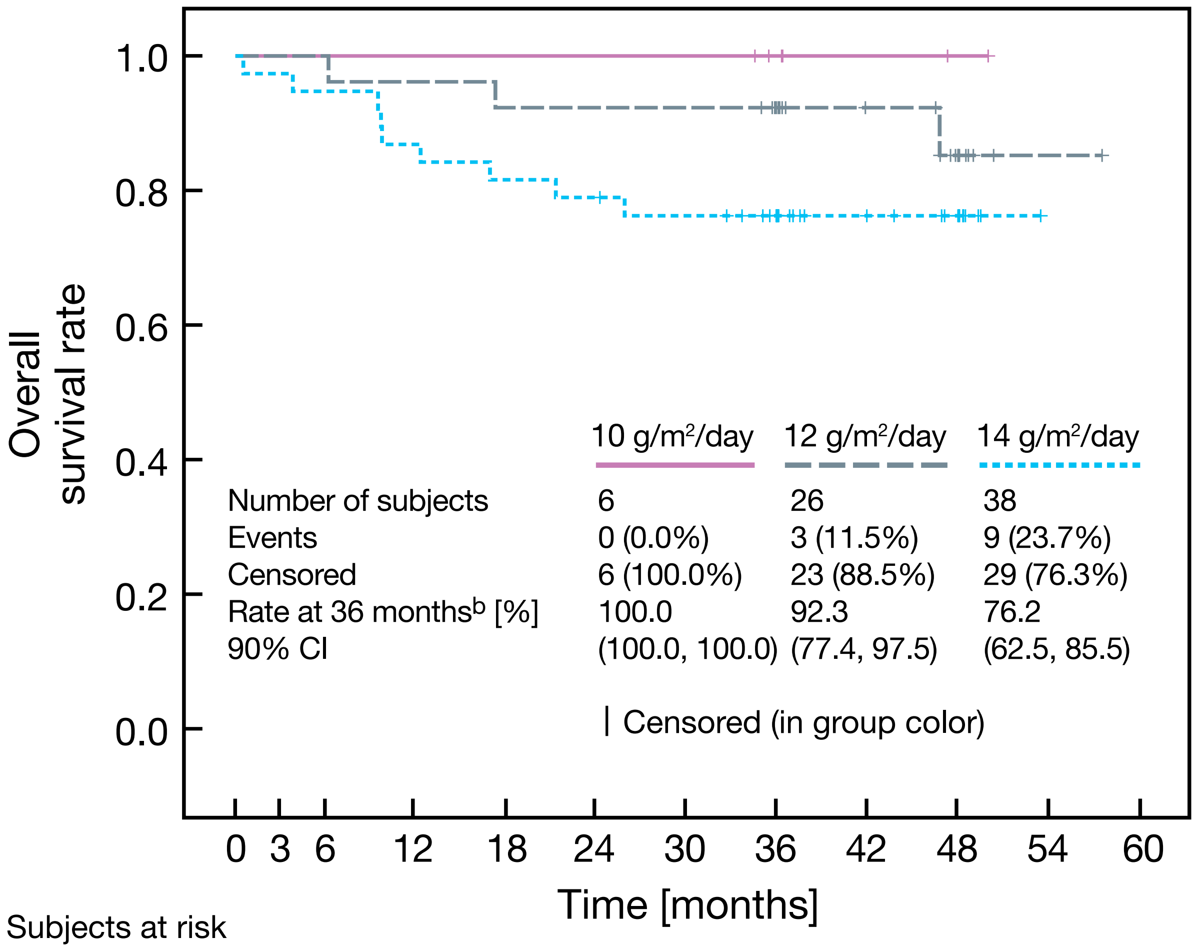

Subjects at risk

10 g/m²/day	6	6	6	6	6	6	6	4	2	1	0	0
12 g/m²/day	26	26	26	25	24	24	24	20	14	7	1	0
14 g/m²/day	38	37	36	33	31	30	28	21	12	8	0	0

[a] Minimum and maximum of observed event times
[b] Based on Kaplan-Meier estimates
