# Supplementary material for: Treosulfan–fludarabine–thiotepa-based conditioning treatment before allogeneic hematopoietic stem cell transplantation for pediatric patients with hematological malignancies
Source: Bone Marrow Transplant. 2020 Mar 20;55(10):1996–2007. doi: 10.1038/s41409-020-0869-6 (PMC7515850; doi:10.1038/s41409-020-0869-6)
Supplement: Supplementary file 3 — Figure S2 [file 41409_2020_869_MOESM3_ESM.pdf]

FIGURE S2 Cumulative incidence of aGvHD grade III - IV by Treosulfan dose

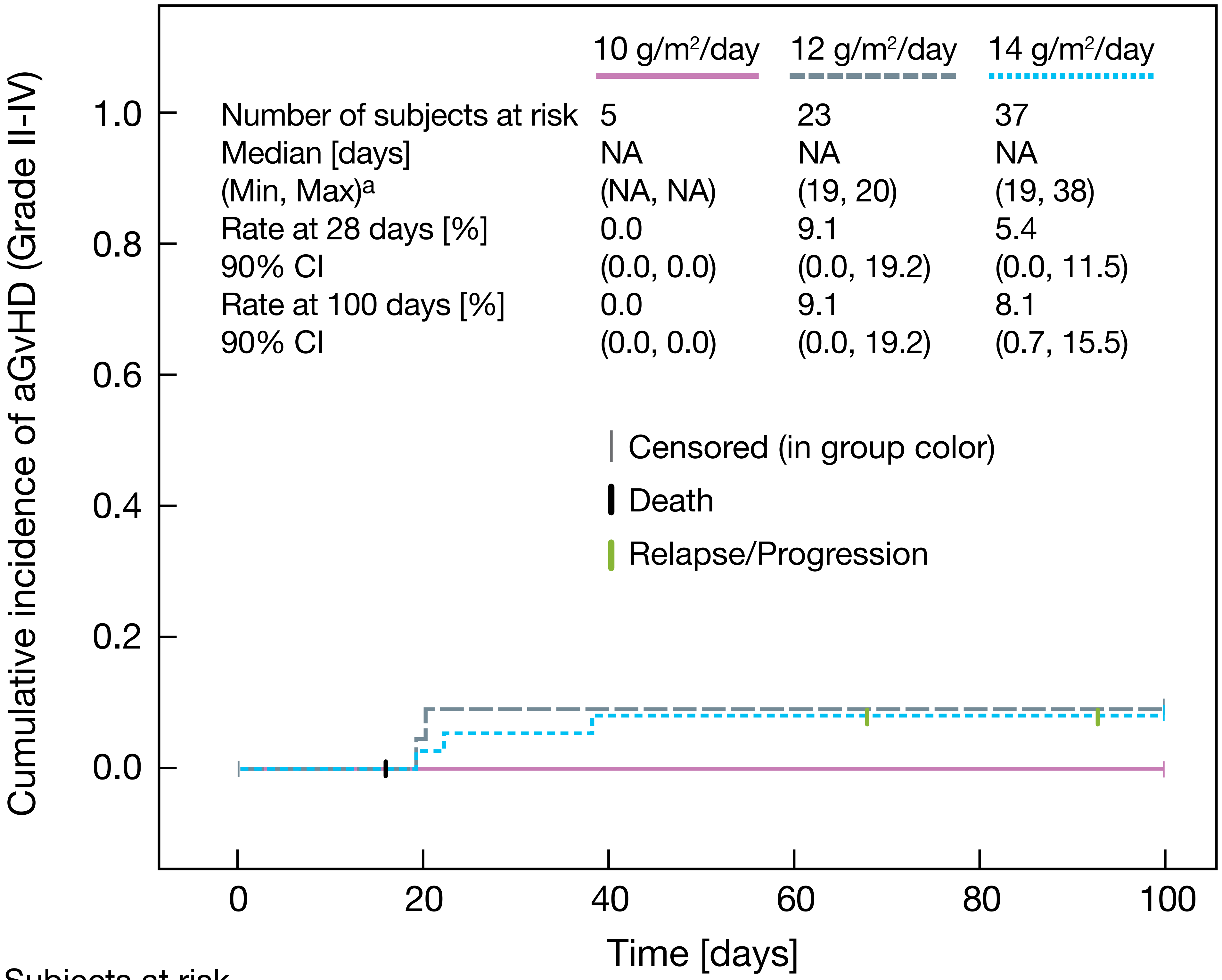

Subjects at risk

10 g/m²/day	5	5	5	5	5	5
12 g/m²/day	23	21	20	20	20	20
14 g/m²/day	37	35	33	33	32	31

[a] Minimum and maximum of observed event times
