# Supplementary material for: Treosulfan–fludarabine–thiotepa-based conditioning treatment before allogeneic hematopoietic stem cell transplantation for pediatric patients with hematological malignancies
Source: Bone Marrow Transplant. 2020 Mar 20;55(10):1996–2007. doi: 10.1038/s41409-020-0869-6 (PMC7515850; doi:10.1038/s41409-020-0869-6)
Supplement: Supplementary file 4 — Figure S3 [file 41409_2020_869_MOESM4_ESM.pdf]

FIGURE S3 **Cumulative incidence of moderate/severe GvHD by Treosulfan dose**

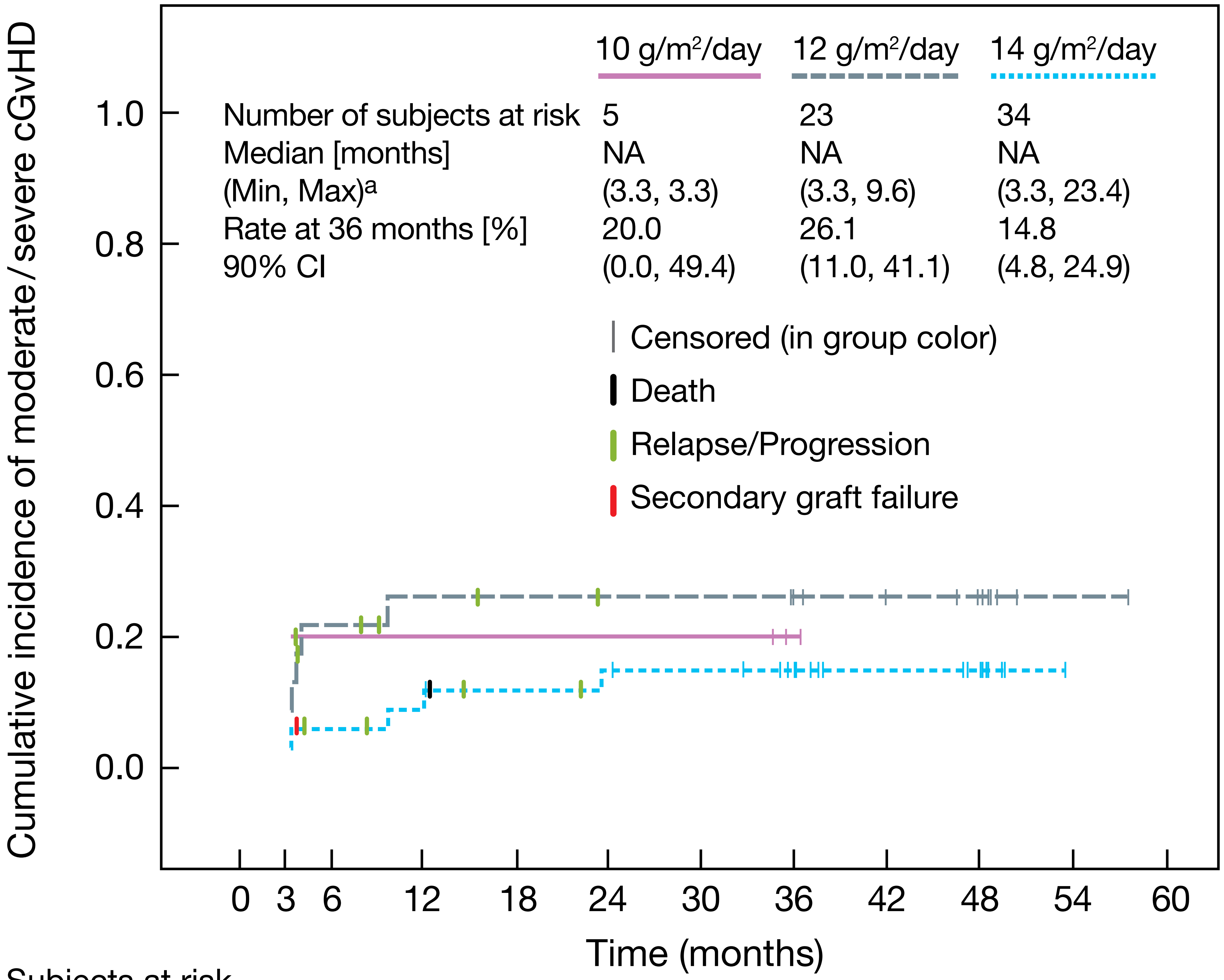

Subjects at risk

10 g/m²/day	3	3	3	3	3	1	0	0	0	0
12 g/m²/day	17	14	13	12	12	10	8	6	1	0
14 g/m²/day	30	27	24	22	21	16	10	7	0	0

Subjects are at risk if they have survived 100 days after end of HSCT without relapse and graft failure. [a] Minimum and maximum of observed event times
